# Pattern of all-causes and cause-specific mortality in an area with progressively declining malaria burden in Korogwe district, north-eastern Tanzania

**DOI:** 10.1186/s12936-018-2240-6

**Published:** 2018-02-27

**Authors:** Daniel P. Challe, Mathias L. Kamugisha, Bruno P. Mmbando, Filbert Francis, Mercy G. Chiduo, Celine I. Mandara, Samuel Gesase, Omari Abdul, Martha M. Lemnge, Deus S. Ishengoma

**Affiliations:** National Institute for Medical Research, Tanga Centre, P.O Box 5004, Tanga, Tanzania

**Keywords:** Pattern, All-causes, Cause-specific mortality, Declining, Malaria

## Abstract

**Background:**

Although death records are useful for planning and monitoring health interventions, such information is limited in most developing countries. Verbal autopsy (VA) interviews are alternatively used to determine causes of death in places without or with incomplete hospital records. This study was conducted to determine all causes and cause-specific mortality in Korogwe health and demographic surveillance system (HDSS) undertaken in Korogwe district, northeastern Tanzania.

**Methods:**

The study was conducted from January 2006 to December 2012 in 14 villages under Korogwe HDSS. Vital events such as births, deaths and migrations were routinely updated quarterly. A standard VA questionnaire was administered to parents/close relatives of the deceased to determine cause of death.

**Results:**

Overall, 1325 deaths of individuals with median age of 46 years were recorded in a population with 170,471.4 person years observed (PY). Crude mortality rate was 7.8 per 1000 PY (95% CI 7.2–8.4) and the highest rate was observed in infants (77.9 per 1000 PY; 95% CI 67.4–90.0). The overall mortality increased between 2006 and 2007, followed by a slight decline up to 2011, with the highest decrease observed in 2012. Causes of deaths were established in 942 (71.1%) deaths and malaria (198 deaths, 21.0%) was the leading cause of death in all age groups except adults (15–59 years). HIV/AIDS (17.6%, n = 365) was the leading cause of death in individuals aged 15–59 years followed by malaria (13.9%) and tuberculosis. Non-communicable diseases (NCDs) including stroke, hypertension, cancer, and cardiac failure caused majority of deaths in elderly (60 years and above) accounting for 37.1% (n = 348) of all deaths, although malaria was the single leading cause of death in this group (16.6%).

**Conclusion:**

The study showed a significant decline of deaths in the Korogwe HDSS site and malaria was the main cause of death in all age groups (except adults, aged 15–59 years) while HIV/AIDS and NCDs were the main causes in adults and elderly, respectively. Further surveillance is required to monitor and document changes in cause-specific mortality as malaria transmission continues to decline in this and other parts of Tanzania.

## Background

All-cause and cause-specific mortality data are useful for estimating the burden of disease as well as planning, monitoring and assessing effectiveness of different health interventions [1]. In order to generate such data, well-established, consistent, systematic, and active vital registration systems are needed [2]. Such systems are limited in most developing countries especially in sub-Saharan Africa (SSA) [3]. Unfortunately, in these countries, about 80% of deaths occur outside health facilities [4] and even the few deaths which occur at health facilities are usually not recorded or the records are inconsistent and unreliable [5]. To complement that, some of these countries adopted and introduced a health and demographic surveillance system (HDSS) which is routinely conducted at specific sites as the platform for generating supportive vital events, including mortality data [6]. Verbal autopsy (VA) method is applied to determine cause-specific mortality within these HDSS sites [7].

The VA is a tool for determining cause-specific mortality, based on responses collected from families and/or caregivers of the deceased from a series of structured questions on the signs and symptoms experienced by the deceased, and their duration [8]. This approach has increasingly become a useful alternative method for estimating cause-specific mortality in areas without vital registration systems [9], despite some technical limitations such as recall bias, questionnaire design, choice of interviewers and respondents, and mechanism for establishing causes of death [10]. The method has been extensively validated and adopted for estimating cause-specific mortality under different settings [11]. It has proved to be useful in estimating all causes, cause-specific as well as seasonal mortality in studies conducted under HDSS [12] and elsewhere [13].

In the majority of SSA countries most deaths in children aged 1–15 years are commonly associated with preventable communicable diseases, with malaria being the leading cause [14]. Other main causes of death in children include diarrhoea and acute respiratory tract infections [15]. For young adults (15–45 years), majority of deaths are due to HIV/AIDS, tuberculosis and malaria [12, 16, 17]. Also, there is a rising epidemic of non-communicable diseases (NCDs) in SSA, particularly chronic conditions such as cardiovascular diseases, diabetes, respiratory diseases and cancers, which contribute about 25% of all deaths [18]. Recent reports have indicated that the number of deaths in Tanzania caused by NCDs and injuries has been increasing, particularly in urban areas [19].

Reducing the burden of disease in developing countries is considered to be one of the most important global tasks, which will reduce poverty and improve general health of communities. Different interventions have been developed to reduce morbidity and mortality attributable to preventable diseases and other causes [20, 21]. However, monitoring such interventions particularly in poor communities is always limited by lack of reliable data to track the trend of morbidity and mortality and thus the impacts of such interventions. Recent studies have shown a dramatic decline of malaria burden in Tanzania and possibly malaria-related mortality due to enhanced malaria control over the past decade [22, 23]. Korogwe district has also experienced a decline of malaria transmission from 78.4% in 2003 in high transmission area [24] to 4.6% between 2011 and 2013 [25]. This has resulted in decrease in the number of patients attending health facilities [26–28]. However, the impact of recent changes in malaria epidemiology on mortality attributable to malaria has received little attention. This study was conducted to determine the pattern of all-cause and cause-specific mortality in an area that has been developed for different malaria interventions [29].

## Methods

### Study area and population

**Fig. 1:**
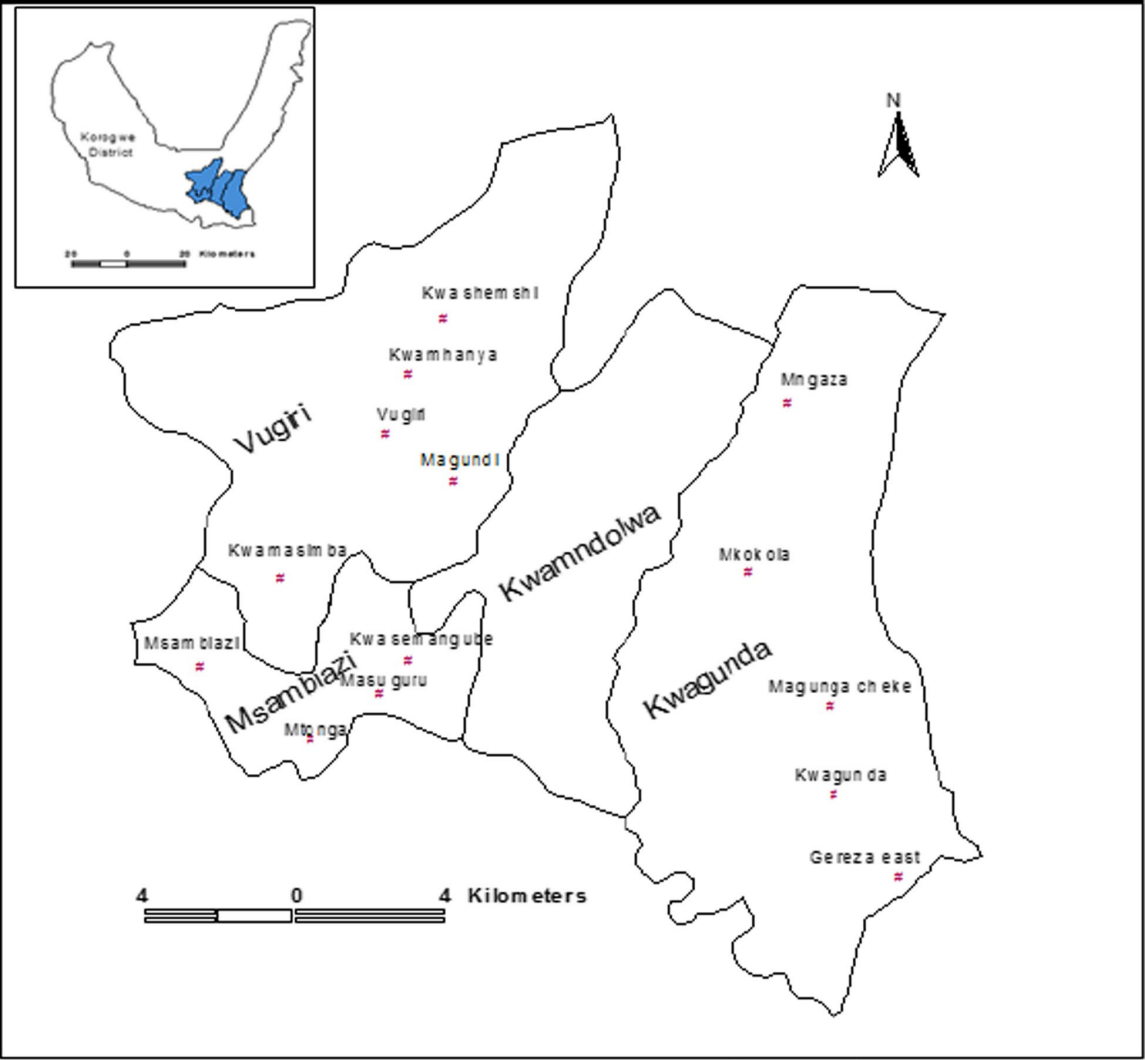
Map showing location of Korogwe district (topleft) and location of the study villages within the wards (centre) (As reproduced from Mmbando et al. [26])

The study was conducted in 14 villages under HDSS in Korogwe district, north-eastern, Tanzania (Fig. 1). Details of the study area have been provided elsewhere [24–26, 29]. Briefly, Korogwe district covers an area of 3756 sq km and it had an estimated total population of 242,038 in 2012; 48.98% were males [30]. The district has four divisions, 20 wards, and 122 villages and it is topographically stratified into lowland and highland zones, with altitudes ranging from 300 to 1200 m above sea level; until recently, lowland areas were hyper/holo-endemic to malaria. Korogwe has 47 dispensaries, four health centres and two hospitals (one owned by the government and one a faith-based organization) [31].

### Study design

The HDSS was implemented in the study villages from January 2006 to December 2012 as previously reported [29]. Through the HDSS, vital events (births, deaths, migrations) were collected and updated three times yearly. For each of the reported deaths, VA interviews were carried out to obtain the probable cause of death by probing for signs and symptoms that were reported or observed before death. The information was collected from guardians or close relatives of the deceased using a VA questionnaire which was adopted from INDEPTH-Network [12].

### Data collection methods

Before initiation of the Korogwe HDSS, a baseline census was conducted by field workers (enumerators) living in the study area. The baseline census was used to obtain background information regarding the study population in order to establish a longitudinal surveillance system. Each household was visited once every 4 months to collect and update information on events that occurred between visits. The HDSS data were recorded in Household Registration Books (HRBs) as described elsewhere [29]. The information collected included deaths, migrations, pregnancies and their outcomes, and household membership. A resident in a HDSS site was defined as any person who had been living in the village for a period of 4 months before registration, women just married and babies born to residents. To maximize chances of capturing deaths and births that occurred in the HDSS, two persons from each village were designated as key informants for reporting the events.

### Ascertaining causes of deaths

A trained field worker conducted a VA interview with a guardian or close relative of the deceased within 2 weeks of the reported death. Two physicians independently reviewed the responses recorded in the questionnaires. A third physician was consulted to review the same VA questionnaires, and in case of discordance, the decision on cause of death was based on two clinicians with similar assessments. A similar assignment by two physicians was considered to be the most likely cause of death. In cases where none of the two physicians had a similar assignment for a particular death, a panel of three clinicians assessed such a case to reach a consensus on the probable cause of death.

### Data management and analysis

The HDSS data were managed using an electronic database, the Household Registration System (HRS) software built in Microsoft Visual FoxPro System [32]. The VA data were double-entered in Microsoft Access database, with consistence checks followed by validation and cleaning. The VA data were linked to the main database (HRS) using unique identifiers of the deceased. Descriptive statistics was used for exploratory analysis and the findings were summarized in text or tabular form showing proportions/rates of different variables. Distributions of categorical variables were compared using χ^2^-test. Study area was stratified into three strata: lowland semi-urban (4 villages), lowland rural (6) and highland rural (4), based on altitude and level of urbanization, which are proxies for malaria transmission [29]. The weighted χ^2^-test was used as the survey estimates to represent population within each strata [33]. Age-specific mortality rates were categorized into 5-year intervals with under-fives further split into two groups (< 1 and 1–4 years), and the maximum age group was 85+ years as according to WHO age standardization of demographic rates [34]. Mortality rate was calculated as the number of deaths per person years observed (PY) [35] from 1 January 2006 to 31 December 2012. The PYs of observation were defined as the follow-up period (baseline or in-migrations) to the point when an event (out-migration or death) occurred. All statistical analyses were carried out using STATA version 13.0 (Stata Corp LP, College Station, TX, USA). A p-value < 0.05 was considered to be significant.

## Results

### Characteristics of the study population and the deceased

Table 1 shows the number of individuals registered and characteristics of the deceased between January 2006 and December 2012. A total of 41,744 individuals [median age of 19 years inter-quartile range (IQR): 9.1–39.5] were registered with 170,471.4 PY, and majority (44.5%, n = 18,588) of these were from lowland urban villages. While, a total of 1325 (3.2%) deaths [median age of 46 years, inter-quartile range (IQR): = 17.7–71.0] also occurred throughout the follow-up period. The median age of the deceased was significantly different among the three strata with lowland rural having the highest median age (p < 0.001). Majority of deaths (54.0%) were males and the proportion was highest in highland rural (males = 62.2%) p = 0.033. Most deaths (39.1%) were adults (15–59 years) while fewer deaths (4.5%) were observed among children aged 5–14 years; infant deaths accounted for 14% of all deaths. Only 30.2% of the deaths occurred at health facilities and this did not differ significantly among the three strata (p = 0.150).

**Table 1:** Characteristics of individuals registered and of the deceased in Korogwe HDSS

Variable	Highland rural	Lowland rural	Lowland urban	Total	χ^2^ p-value
Number registered (PYL)	8076 (36,274.0)	15,080 (59,912.1)	18,588 (74,285.3)	41,744 (170,471.4)	
Median age of the population-years, (IQR)	18 (9.0–39.5)	19 (8.8–41.5)	19 (9.4–38.5)	19 (9.1–39.5)	< 0.001
Number of deaths	209 (15.8)	585 (44.1)	531 (40.1)	1325 (100.0)	
Median age of deceased-years, (IQR)	43 (11.7–67.6)	49 (12.8–72.6)	46 (24.7–69.8)	46 (17.7–71.0)	< 0.001
Gender, n (%)					
Male	130 (62.2)	305 (52.1)	280 (52.7)	715 (54.0)	
Female	79 (37.8)	280 (47.9)	251 (47.3)	610 (46.0)	0.033
Age group, n (%)					
Infants (< 1 year)	30 (14.3)	92 (15.7)	62 (11.7)	184 (14.0)	
Under fives (1–4 years)	15 (7.2)	33 (5.6)	29 (5.5)	77 (5.8)	
Children (5–14 years)	10 (4.8)	26 (4.5)	24 (4.5)	60 (4.5)	
Adults (15–59 years)	82 (39.2)	203 (34.7)	233 (43.9)	518 (39.1)	
Elders (60+ years)	72 (34.5)	231 (39.5)	183 (34.4)	486 (36.6)	0.133
Place of death, n (%)					
Home^a^	176 (84.2)	434 (74.2)	315 (59.3)	925 (69.8)	
Heath facility	33 (15.8)	151 (25.8)	216 (40.7)	400 (30.2)	0.150

^a^Includes deaths which occurred at home and places other than health facilities

### Trends of mortality rates in the 14 villages under Korogwe HDSS

**Fig. 2:**
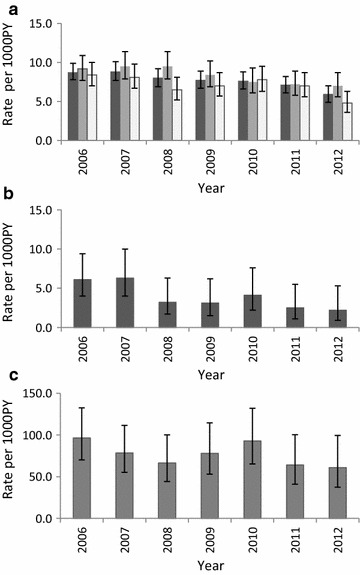
Trends and pattern of mortality rates in Korogwe HDSS from 2006 to 2012. **a** Is for overall mortality rates stratified by gender; the black bar represents overall mortality rates, the grey bar represents male mortality rates, colourless bar represents female mortality rates. **b** Represents under-fives mortality rates; **c** represents infant mortality rates

The overall mortality rate was 7.8 per 1000 PY (95% CI 7.2–8.4) with infants having a higher rate (77.9 per 1000 PY; 95% CI 67.4–90.0). The mortality rate was higher in males (8.4 per 1000 PY; 95% CI 7.8–9.0) than in females (7.2; 95% CI 6.6–7.7). The risk of dying for males was 15% higher than for females (p = 0.004). Compared to highland rural, the risk of dying was significantly higher in both lowland rural (RR = 1.69, 95% CI 1.44–1.98, p ≤ 0.001) and lowlands urban (RR = 1.23, 95% CI 1.06–1.45, p ≤ 0.000) (Table 2). Despite a decline in mortality rates in the Korogwe HDSS, males had consistently higher mortality rates than females except in 2010. The overall mortality increased between 2006 and 2007 followed by a slight decline up to 2011, with the highest decrease observed in 2012 (Fig. 2a). A similar trend was observed among under-fives and the decline in mortality rates were the highest in this group, with a decrease of over 65.1% in the entire period despite a slight increase in 2007 (Fig. 2b). For infants, a decline in mortality rates was also observed but the overall reduction was low compared to under-fives (Fig. 2c).

**Table 2:** Mortality rates in 14 villages of Korogwe HDSS

Variable	Deaths				
	Number of deaths, n (%)	PY	Mortality rate per 1000 PY (95% CI)	Rate ratio (95% CI)	χ^2^ p-value
Age group					
Infants (< 1 year)	184 (13.9)	2361.2	77.9 (67.4–90.0)	1	
Under-fives (1–4 years)	77 (5.8)	18,769.6	4.1 (3.3–5.1)	0.55 (0.04–0.07)	< 0.001
Children (5–14 years)	60 (4.5)	46,926.8	1.3 (1.0–1.6)	0.02 (0.01–0.02)	< 0.001
Adults (15–59 years)	518 (39.1)	85,888.4	6.0 (5.5–6.6)	0.08 (0.07–0.10)	< 0.001
Elderly (60+ years)	486 (36.7)	16,525.4	29.4 (26.9–32.1)	0.39 (0.33–0.47)	< 0.001
Gender					
Male	715 (54.0)	85,313.3	8.4 (7.8–9.0)	1	
Female	610 (46.0)	85,158.1	7.2 (6.6–7.8)	0.85 (0.77–0.95)	0.004
Strata					
Highland rural	209 (15.8)	36,274.0	5.8 (5.0–6.6)	1	
Lowland rural	585 (44.1)	59,912.1	9.8 (9.0–10.6)	1.69 (1.44–1.98)	< 0.001
Lowland urban	531 (40.1)	74,285.3	7.1 (6.6–7.8)	1.24 (1.06–1.45)	0.009
Total	1325	170,471.4	7.8 (7.4–8.2)

### Cause-specific mortality

**Fig. 3:**
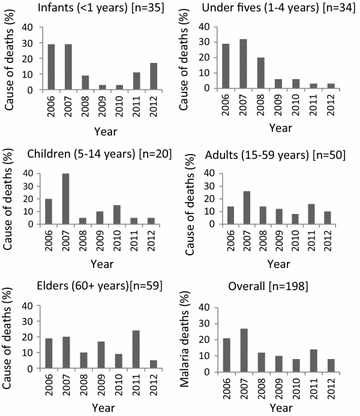
Pattern of malaria-specific deaths in Korogwe HDSS from 2006 to 2012

Causes of death were established in 942 (71.1%) individuals, as presented in Table 3. Malaria was the leading cause of death in all age groups except in individuals aged 15–59 years, and it accounted for 21.0% (n = 198) of all causes. Among the different age groups, the proportion of deaths caused by malaria ranged from 13.9% in individuals aged 15–49 years to 59.6% in children aged 1–4 years (Table 3). The pattern of malaria deaths in all age groups showed a peak in 2007 with the exception of elderly (≥ 60 years) which had peak malaria deaths in 2011. Among infants, malaria deaths dropped markedly from 28.6% in 2006 to 2.9% in 2009 but increased to 17.1% in 2012. Generally, malaria deaths declined significantly from 2006 to 2012 with a pronounced decline among infants and under-fives (Fig. 3). Among elderly (≥ 60 years), malaria-specific deaths did not show a declining pattern as observed in other age groups but was lower in 2008, 2010 and 2012 (Fig. 3).

**Table 3:** Causes of death by age groups in Korogwe HDSS from 2006 to 2012

Cause of death	Age groups				
	Infants (< 1 year)	Under-fives (1–4 years)	Children (5–14 years)	Adults (15–59 years)	Elderly (60+ years)
	n (%)^[rank]^	n (%)^[rank]^	n (%)^[rank]^	n (%)^[rank]^	n (%)^[rank]^
Malaria	35 (28.2)^[1]^	34 (59.6)^[1]^	20 (41.7)^[1]^	49 (13.9)^[2]^	60 (16.6)^[1]^
Pneumonia	20 (16.1)^[2]^	7 (12.2)^[2]^	0 (0)	0 (0)	0 (0)
Sepsis	13 (10.5)^[3]^	0 (0)	0 (0)	0 (0)	0 (0)
BA	12 (9.7)^[4]^	0 (0)	0 (0)	0 (0)	0 (0)
Premature	8 (6.5)^[5]^	0 (0)	0 (0)	0 (0)	0 (0)
SBF	4 (3.2)^[6]^	0 (0)	0 (0)	0 (0)	0 (0)
HIV/AIDS	0 (0)	3 (5.3)^[3]^	0 (0)	62 (17.6)^[1]^	0 (0)
Accident	0 (0)	3 (5.3)^[4]^	2 (4.2)^[4]^	16 (4.6)^[5]^	0 (0)
Malnutrition	0 (0)	2 (3.5)^[5]^	2 (4.2)^[5]^	0 (0)	0 (0)
Diarrhoea	0 (0)	2 (3.5)^[6]^	0 (0)	0 (0)	0 (0)
Epilepsy	0 (0)	0 (0)	5 (10.3)^[2]^	0 (0)	0 (0)
Anaemia	0 (0)	0 (0)	3 (6.2)^[3]^	0 (0)	0 (0)
Cancer	0 (0)	0 (0)	2 (4.2)^[6]^	19 (5.4)^[4]^	28 (7.8)^[4]^
Tuberculosis	0 (0)	0 (0)	0 (0)	34 (9.7)^[3]^	25 (6.9)^[6]^
HF	0 (0)	0 (0)	0 (0)	11 (3.1)^[6]^	27 (7.5)^[5]^
Stroke	0 (0)	0 (0)	0 (0)	0 (0)	41 (11.4)^[2]^
HTN	0 (0)	0 (0)	0 (0)	0 (0)	33 (9.1)^[3]^
Others	16 (12.9)^[7]^	3 (5.3)^[7]^	7 (14.5)^[7]^	121 (31.2)^[7]^	92 (28.5)^[7]^
Undetermined	16 (12.9)^[8]^	3 (5.3)^[8]^	7 (14.5)^[8]^	53 (14.5)^[8]^	44 (12.1)^[8]^
Total	124 (100.0)	57 (100.0)	48 (100.0)	365 (100.0)	348 (100.0)

BA, birth asphyxia; HF, heart failure; HTN, hypertension; Malnut, malnutrition; SBF, spina bifida

HIV/AIDS was the main cause of death (17.6%, n = 62) among adults aged 15–59 years followed by malaria (13.9%), tuberculosis (9.7%), and cancer (5.4%). Other major causes of death in infants were pneumonia and sepsis, while in under-fives, pneumonia, HIV/AIDS and accidents were the leading causes of death after malaria. In children (5–14 years), other leading causes of death were epilepsy (10.3%) and anaemia (6.2%). Among elderly (≥ 60 years), NCDs, including stroke, hypertension, cancer, and heart failure were the main causes of death after malaria, followed by tuberculosis (6.9%), Table 3.

## Discussion

This study assessed and summarized the trends and patterns of all-cause and cause-specific mortality in Korogwe HDSS. Few studies in Tanzania have been conducted to investigate cause-specific mortality with respect to different malaria transmission intensity and also document changes in malaria deaths, particularly following a progressive decline of malaria burden in recent years [36]. Over the 7-year period, the study observed significant number of deaths and cause-specific mortality. Overall, mortality rates in the area were lower compared to those previously reported in other parts of Tanzania [37, 38] and sub-Saharan Africa [39–42]. The main reasons for declining trends in mortality, apart from the decline in malaria burden, could include socio-economic development, improved health services, such as the establishment of primary health care, provision of preventive services and immunization.

This study showed that the majority of deaths, even those involving infants and under-fives, occurred at home, which might be due to poor utilization of available health facilities in the study area. However the proportion of deaths which occurred out of health facilities in this site was lower (by approximately 10%) compared to the proportion previously reported in Nairobi HDSS, Kenya [4]. Similarly, a majority of deaths had occurred at home, as shown by other studies conducted in Muheza district (72.4%) [43] and in Kenya (87.0%) [17]. All villages involved in this study either have a dispensary or are within 5 km of the nearest health facility. The reason for failure of community members to utilize health services provided by these facilities, even in the terminal stages of a deceased’s illness, is not clearly known. However, other studies observed that place of residence, either urban or rural, contributed to home deaths [44].

The increase in all-cause mortality trends (overall and in under-fives mortality) and malaria-specific mortality between 2006 and 2007 could probably be due to abnormal and prolonged short rains towards the end of 2006. Previous malaria incidence studies conducted in the study area [26] showed an increase in malaria incidence during the same period as a result of high rainfall in 2006. This suggests that overall trends of mortality were highly correlated to malaria-specific mortality and the general level of malaria transmission in the area. Although this might need to be further assessed, the high correlation of malaria morbidity with the general trend of mortality in Korogwe indicates that a continued decline of malaria transmission will most likely lead to a significant decline of mortality in the population. According to WHO, the reduction of malaria mortality rates among under-fives in Africa has led to a rise in life expectancy at birth for 12% from 50.6 years in 2000 to 60 years in 2015 [23]. Another study showed that a decline in malaria incidence occurred together with that in invasive bacterial infections [45] and this could possibly account for the decline of all-cause mortality that occurred concurrently with malaria-specific deaths.

Although overall mortality rates showed a slight decline, the rates were higher among males compared to females. The difference in mortality among males and females has been described elsewhere as a mixture of biological difference, behavioural factors, such as more smoking in males and socio-economic roles whereby males tend to be employed in more dangerous, harmful, stressful, or difficult occupations than females [46, 47]. Vulnerability of non-immune individuals, including infants, to infectious diseases might be the reason for sustained high mortality in infants compared to under-fives. However, reasons for persistently high mortality among infants are not clearly known. Since mortality data are an important indicator of performance and quality of health services in a given country/region, more studies are needed to determine the reasons for the persistently high rate of deaths among infants.

Communicable diseases were responsible for the majority of deaths, whereby malaria was the leading cause in all age groups except for individuals aged 15–49 years. Findings from other sites in SSA with high malaria transmission intensity have reported high malaria mortality among infants and children up to 15 years of age and a significant contribution to adult deaths [13, 16, 24, 48]. Another main cause of both infant and under-fives mortality was pneumonia while sepsis contributed almost 10% of infant deaths, as observed in other sites [49]. However, different patterns have been reported in western African sites where diarrhoea and upper respiratory infections were among the main causes of mortality in children and other age groups [16, 50]. This study showed that HIV/AIDS caused a larger proportion of deaths among adults aged 15–59 years and this is consistent with findings reported from other sites in SSA [17, 51, 52]. Tuberculosis was also one of the main causes of deaths in adults above 15 years and this can be linked to its co-morbidity with HIV/AIDS [12, 53]. Unlike previous reports from other areas in SSA [12, 17], NCDs (stroke, hypertension, cancer, cardiac failure) caused more than one-third of all deaths in elderly (≥ 60 years old). This may indicate an increase in NCD-related mortality in rural and semi-urban villages, which is in line with that previously reported in urban and prosperous rural communities in Tanzania [54]. Further surveillance will be required to track the pattern and trends of NCD morbidity and mortality in order to devise and implement appropriate control interventions, particularly with the current pattern of declining burden of malaria and other infectious diseases.

Previous malaria epidemiological studies, both community and hospital based in Korogwe showed a decrease of malaria transmission in the area [24, 55–57]. Entomological studies has also reported decline of populations of malaria vectors, which may have resulted into low malaria infection and hence the decline in malaria burden and deaths [58]. In general, the results showed the decrease in malaria mortality in the study area from 2006 to 2012, a similar pattern as reported in other areas of Tanzania [59] as well as other malaria-endemic areas [60].

Data generated using VA are usually faced with inherent limitations associated with methods that include recall bias, questionnaire design, choice of interviewers and respondents, and mechanism for establishing causes of death. However, the study managed to reduce the recall bias by conducting VA assessment within 2 weeks after death. The INDEPTH VA questionnaire was used to collect information on the possible causes of death to ensure that it captured standardized information which is commonly required for this type of analysis. A trained fieldworker collected the data by conducting interviews according to standard operating procedures and hence reduced the choice of interviewers’ variability and respondents’ limitations. Two physicians assessed the VA questionnaires to establish the causes of death and a third physician helped to resolve any discordant assignment, and this helped to minimize and reduce the chances of wrong assignment of causes of death. Despite these limitations, the data presents important information on the trends of deaths, cause-specific mortality and trends of malaria deaths in Korogwe HDSS. The interpretation and generalization of the findings might be limited to this site.

## Conclusion

This study showed a significant decline of deaths in the Korogwe HDSS site and malaria was the main cause of death in all ages except individuals aged 15–59 years, with a declining trend over the study period. Most of the adult deaths (among those aged 15–59 years) were mainly caused by HIV/AIDS, while malaria and NCDs (stroke, hypertension, cancer, cardiac failure) caused the majority of deaths in elderly (aged 60 years and above). Although control interventions aiming at reducing the burden of communicable diseases in the study area need to be sustained, specific interventions targeting NCDs among adults and elderly are also urgently required.
